# What do we know about evidence-informed priority setting processes to set population-level health-research agendas: an overview of reviews

**DOI:** 10.1186/s42269-021-00687-8

**Published:** 2022-01-06

**Authors:** Audrey Tan, Sumanth Kumbagere Nagraj, Mona Nasser, Tarang Sharma, Tanja Kuchenmüller

**Affiliations:** 1 Office of the Vice-Provost (Research, Innovation and Global Engagement), University College London, 2 Taviton Street, London, WC1H 0BT UK; 2 Faculty of Health: Medicine, Dentistry and Human Sciences, University of Plymouth, The John Bull Building, Research Way, Plymouth, PL6 8BU Devon UK; 3 WHO Regional Office for Europe, UN City, Marmorvej 51, 2100 Copenhagen, Denmark

**Keywords:** Research priority setting, Overview of reviews, Systematic review, National health research system, Research agenda, Research funding, Resource allocation

## Abstract

**Background:**

This overview aimed to synthesize existing systematic reviews to produce a draft framework of evidence-informed health priority setting that supports countries in identifying appropriate steps and methods when developing and implementing national research agendas.

**Main body:**

We searched Ovid MEDLINE^®^ and the WHO Institutional Repository for Information Sharing from 2010 to 2020 for critical or systematic reviews that evaluated research priority setting exercises. We adapted the AMSTAR checklist to assess the quality of included reviews and used adapted frameworks for data extraction and analysis. The search resulted in 2395 titles, of which 31 were included. Populations included in the reviews typically involved patients, families and carers, researchers, clinicians, policymakers and research funders. The topics covered in the reviews varied from specific diseases or conditions, approaches for healthcare practice or research priority setting methods itself. All the included systematic reviews were of low or critically low quality. The studies were thematically grouped based on their main focus: identifying and engaging with stakeholders; methods; context; and health area.

**Conclusion:**

Our overview of reviews has reconfirmed aspects of existing frameworks, but has also identified new concepts for countries to consider while developing their national research agendas. We propose a preliminary framework for consideration that highlights four key phases: (1) preparatory, (2) priority setting, (3) follow-up phase and (4) sustainability phase, which have thirteen sub-domains to consider.

## Background

In recent years, an evidence-based approach has not only become a cornerstone for informing health practice policy, but it has also become a key part of informing decisions on how to conduct and organize research. This includes how to set priorities for research, which is a political and social process that is informed by the views and experiences of the stakeholders along with available data and information. The research priority setting approach depends on the context, stakeholders and why an organization is conducting the priority setting exercise in the first place. Due to its complexity, more recent studies focus on individual elements or steps of the priority setting process and how these can lead to certain outcomes rather than evaluating the broader priority setting process.

The past two decades have also seen an increase in efforts to develop better stakeholder engagement, especially with patients, to set research priorities (Stewart et al. 2011). This growing movement has been accompanied by a proliferation of primary literature on the conduct, evaluation, and reporting of research priority setting, as well as a subsequent increase of systematic reviews focusing on those areas. There are also numerous guidance documents and manuals on how to set priorities for research (World Health Organization 2020a; Ghaffar et al. 2009; McGettigan and Henry 2011).

While the documents, such as the World Health Organization (WHO) guidance (World Health Organization 2020a), draw on existing research priority setting experiences (tacit knowledge) and examples of good practice, the methods used in the development of these documents are not always inclusive of a systematic evaluation of the literature. Systematic reviews to date have consolidated published priority setting exercises across a variety of fields and disciplines, such as a systematic review of research priority setting in childhood chronic disease (Odgers et al. 2018) and a systematic review on research priority setting exercises in Zambia (Chanda-Kapata et al. 2016). Recent systematic reviews have also acknowledged the high degree of variability in methods used for collecting priority topics and have expressed the need for more standardized methods (Roche et al. 2021).

The COVID-19 crisis has placed enormous pressure on healthcare systems, health research systems and policymakers. These pressures underscore the importance and need, more so now than ever, of consolidated frameworks and processes for performing and evaluating research priority setting exercises, particularly for use at a system and country level. To facilitate the widespread use of evidence, information and research, a systems approach in health research is critical to ensure effective coordination and knowledge translation (Hanney et al. 2020). During the COVID-19 crisis and indeed, in the aftermath, when countries emerge from the acute stages of managing outbreaks, frameworks are needed to facilitate decision makers in allocating limited time, funding and resources to the most relevant and pressing research topics (World Health Organization 2020b).

Systematic reviews primarily address a focused research question. Given that research priority setting processes contain multiple components and stages, a single systematic review is unlikely to be sufficiently comprehensive to assess all these components. Overviews of reviews (hereafter referred to as overviews) “compile data from multiple systematic reviews to provide a single synthesis of relevant evidence for decision making” (Pollock et al. 2016) and can therefore be more accessible to decision makers. Overviews can also avoid uncertainty created by conflicting conclusions from different reviews, which may vary in scope and quality. While the volume of reviews assessing different aspects of research priority setting continues to grow, there is an increasing need amongst policymakers for an overarching overview of reviews that consolidates existing reviews and identifies which practices can be translated into policy, and which areas require further research. Therefore, this overview aimed to synthesize existing systematic reviews to produce a framework for evidence-informed health priority setting processes that could be considered by countries to support the development of national public health research agendas.

## Main text

### Protocol and registration

The protocol of this overview was drafted in accordance with the Preferred Reporting Items for Systematic Reviews and Meta-Analysis Protocols (PRISMA-P) checklist (Moher et al. 2015). As there is no defined guidance on how to conduct systematic reviews on research priority setting methods or for overviews of methodological reviews, we utilized an adapted approach of the methodology developed for performing overviews of clinical topics (Pollock et al. 2016). The complete methods included in the protocol are described in the registered protocol on Open Science Framework (https://osf.io/necm9) and are summarized below.

### Eligibility criteria

Our primary objective was to identify methods for setting priorities in research that inform population level health-related research agenda (*context*). However, not all studies clearly report whether they focus on population-level or patient-related issues. Thus, for the purposes of our review, we did not restrict our studies based on *target population* and included all health-related priorities. The *core concept* examined is any critical or systematic review that evaluates research priority setting exercises that either (1) involve stakeholders; or (2) utilize a transparent and data-driven approach to analyse the process. Though preferably we identified priority setting exercises that contain both elements. Stakeholders are defined as patients, caregivers, general community, health professionals, researchers, policymakers, non-governmental organizations, government, industry, as well as specific groups, including vulnerable and marginalized populations (Tong et al. 2019).

### Types of studies

We included any critical or systematic review on health research priority setting that focused on a specific topic or methodology aspect, for example, stakeholder engagement in setting priorities. A critical review is defined as a review that has “extensively researched the literature and critically evaluated its quality” (Grant and Booth 2009). Given the rapid nature of this review, we only considered published, full-text studies. Abstracts, editorials, letters, commentaries, opinion pieces and case studies were excluded. Studies were limited to English language and those published from 2010 to 2020. This date range was chosen as systematic reviews before this period were expected to be too out of date for the purposes of our overview and would not be expected to reflect current best practice. As there is currently no clear definition outlining the standards for high quality systematic reviews in setting priorities for research, we considered any critical or systematic review that transparently defined how they searched and selected included studies.

Some of these reviews have a more descriptive approach to synthesizing reports of research priority setting, while others have evaluated and compared the methods used. We included all these types.

### Types of outcomes

We expected reviews to measure and evaluate the success or effectiveness of a research priority setting exercise through either a process evaluation (focusing on whether the research priority setting activities were implemented as intended and resulted in certain outputs) or as an outcome evaluation (focusing on assessing the progress in the outcomes that the research priority setting activities are trying to address) (Nasser et al. 2013). We included outcomes of either type.

### Data sources and search for studies

The review team identified search strategies utilized in relevant systematic reviews and piloted the strategy in Ovid MEDLINE^®^. The final search was peer-reviewed using the Peer-Review of Electronic Search Strategies (PRESS) guideline (McGowan et al. 2016) and the full electronic search strategy for MEDLINE can be found in “Appendix A”. We searched Ovid MEDLINE^®^ and the WHO Institutional Repository for Information Sharing (IRIS). Vocabulary and syntax were adjusted across databases. Strategies utilized a combination of controlled vocabulary (e.g. review) and keywords (e.g. priority setting).

We adopted a pragmatic approach for conducting the search. Ovid MEDLINE^®^ and the WHO Institutional Repository for Information Sharing (IRIS) were searched in May and June 2021, respectively. We expected that most of the research priority setting studies would be identified through this search. Vocabulary and syntax were adjusted across databases. Publications only after 2010 were considered using appropriate limit functions. Strategies utilized a combination of controlled vocabulary (e.g. review) and keywords (e.g. priority setting).

Additionally, we contacted methods experts in the WHO and Cochrane to identify any additional relevant material and hand-searched the reference lists of included studies.

### Data management

The final search results the systematic reviews from all databases were imported into Reference Manager v12 (RefMan 2011), and duplicates were removed by (MN). De-duplicated results were imported into Rayyan, an online systematic review management software (Ouzzani et al. 2016).

### Study selection

De-duplicated titles from Rayyan were then imported to Covidence for study selection. Title and abstract screening was performed by two authors (AT, SKN). To ensure a high level of agreement, AT and SKN performed a pilot screening of 20 titles. The minimum threshold agreement of 80% was met (90% agreement), so AT and SKN then divided the task of screening the records (non-blinded, single screening).

Full text screening was performed by two authors (AT, SKN). To ensure a high level of agreement, AT and SKN performed a pilot screening of 20 titles. The minimum threshold agreement of 80% was met (90% agreement), so AT and SKN then divided the task of screening the full texts (non-blinded, single screening).

If the same primary study was reported in multiple systematic reviews, the systematic reviews and data from the individual study were included in our synthesis regardless. While the issue of overlapping systematic reviews is a methodological concern for overviews of clinical systematic reviews, this issue is not as applicable for our review as we did not conduct meta-analyses and thus not affected by the issue of double counting of primary studies. If the same primary study is reported in multiple systematic reviews, the systematic reviews and data from the individual study was included in our synthesis regardless of whether it has been included in more than one systematic review. However, we performed a holistic assessment of overlapping systematic reviews to view how the interpretations and conclusions varied and whether this affected our own conclusions.

### Data extraction

We drafted the data-extraction form using MS Excel 2016, based on consolidated reporting items identified in the REporting guideline for PRIority SEtting of health research (REPRISE) checklist, which contains ten domains covering: context and scope, governance and team, framework for priority setting, stakeholders/participants, identification and collection of priorities, prioritization of research topics, output, evaluation and feedback, translation and implementation, and funding and conflict of interest (Tong et al. 2019). Any assumptions or definitions are explained in the relevant sections of this review. Two reviewers (AT, SKN) pilot-tested the data-extraction template on one study and discussed the results with the wider team. AT and SKN worked in tandem to extract the data, discussed the results and continuously updated the data-extraction form in an iterative process (Levac et al. 2010). A list of major changes to the data-extraction form is provided as an appendix.

## Quality assessment and synthesis

### Quality assessment

The A MeaSurement Tool to Assess systematic Reviews 2 (AMSTAR 2) checklist assesses the quality of systematic reviews and is commonly used to assess the quality of systematic reviews on effectiveness of interventions (Shea et al. 2017). However, this checklist is less relevant to systematic reviews of priority setting methods as the structure of the research is different. For the purposes of this review, we developed an adapted version of the AMSTAR 2 checklist with guidance that was relevant for priority setting methods (see “Appendix B”). While the original 16 items were maintained, the questions were adapted so that the research priority setting method, as opposed to the intervention, was the focus. We built the adapted checklist based on the experience of the authors in the field, items identified in the REPRISE reporting guideline (Tong et al. 2019) and evaluation frameworks for priority setting (Nasser et al. 2020). The method for determining the overall confidence followed that of the standard AMSTAR 2 guidance with ratings from high (zero or one non-critical weakness), moderate (more than one non-critical weakness), low (one critical weakness with or without non-critical weaknesses) and critically low (more than one critical weakness with or without non-critical weaknesses).

### Data synthesis

To manage the heterogeneity (in both focus and methods used) in the reviews, we first iteratively organized studies based on their main focus (i.e. engaging with stakeholders; methods; context; or health area). These themes were then further organized by geographical area and context (i.e. global, high-income countries [HICs], low- and middle-income countries [LMICs], specific countries/regions or no geographical specification) in recognition that the complexity of a priority setting process is dependent on the resources available. Studies were thematically analysed within these subthemes. We used the World Bank’s definition of HICs or LMICs as this was also the practice of many of the included studies. The synthesis was then used to develop an evidence-informed framework for research priority setting, which built on guidance included in four of the most recent, previously published frameworks (Fadlallah et al. 2020; Nasser et al. 2020; Viergever et al. 2010; World Health Organization 2020a).

## Results

### Results of search

**Fig. 1:**
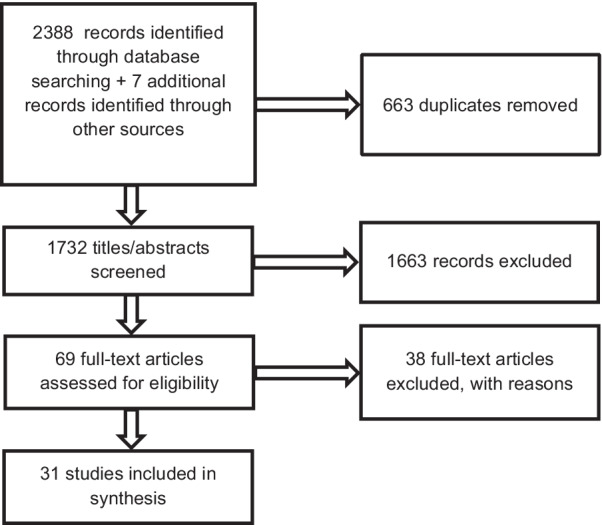
PRISMA flowchart of study selection

The PRISMA flowchart of study selection (Fig. 1) shows the selection process of 2388 records identified from the literature search and an additional seven records that were identified through hand searching the bibliographies of included studies and consulting experts in the field. After duplicates were removed, two review authors (AT and SKN) excluded 1732 records at the title and abstract screening stage. AT and SKN assessed 69 full-text articles for eligibility and included 31 studies in this review. The reasons for exclusion are listed in “Appendix C” and included: duplicate studies, the core concept not focusing on research priority setting exercises or an ineligible study design.

The characteristics of included studies (Table 1) provides an overview of the studies’ characteristics. Of the 31 systematic reviews included in this overview, sixteen reviews (Alqahtani et al. 2021; Bragge et al. 2015; El-Harakeh et al. 2019; El-Harakeh et al. 2020; Fadallah et al. 2020; Garcia et al. 2017; Graham et al. 2020; Roche et al. 2021; Rudan et al. 2017; Rylance et al. 2010; Stewart et al. 2011; Tong et al. 2015; Tong et al. 2017; Viergever et al. 2010; Wade et al. 2021; Yoshida 2016) had a global scope. Additionally, five reviews (Cadorin et al. 2020; Hasson et al. 2020; Hawwash et al. 2018; Manafo et al. 2018; Mörelius et al., 2020) specifically focused on HICs, two reviews (McGregor et al. 2014; Tomlinson et al. 2011) focused on LMICs, five reviews (Badakhshan et al. 2018; Booth et al. 2018; Bryant et al. 2014; Garcia et al. 2015; Reveiz et al. 2013) focused on specific countries or regions of countries and three (Bourne et al. 2018; Odgers et al. 2018; Terry et al. 2018) did not specify geographic settings.

**Table 1:** Characteristics of included studies

Study ID	Objective	Scope of review	Stakeholder population
Alqahtani et al. (2021)	‘To identify potential future areas of development and research in mobility-assistive technology’ (p. 362)	Global	People with lived experience, healthcare professionals
Badakhshan et al. (2018)	‘To evaluate the quality of the priority setting reports about health research in Iran’ (p. 753)	Iranian health research organizations	HCPs, researchers, policymakers
Booth et al. (2018)	To map research priorities identified from existing research prioritization exercises relevant to infants, children and young people with life-limiting conditions’ (p. 1552)	Health research organizations in OECD countries	Children and young people, parents/carers, HCPs, third sector workers, researchers
Bourne et al. (2018)	‘To describe research methods used in priority-setting exercises for MSK conditions and synthesize the priorities identified’ (p. 1)	No setting specifications	Patients/consumers, clinicians, researchers, policymakers and/or funders
Bragge et al. (2015)	‘To gain an overview of the volume, nature and findings of studies regarding priorities for spinal cord injury research’ (p. 714)	Global	Patients, patient representatives, families and carers; researchers; clinicians; policymakers; research funders; and representatives of healthcare organizations
Bryant et al. (2014)	‘To examine methods, models and frameworks used to set health research priorities’ (p. 1)	Priority setting exercises from North America, Europe and Australia, and New Zealand	HCPs, researchers, policymakers, consumers, educators
Cadorin et al. (2020)	‘To describe cancer nurses and patients’ main research priorities and describe their development over time’ (p. 238)	HICs	Patients diagnosed with cancer or nurses involved in their care
El-Harakeh et al. (2019)	‘To identify and describe prioritization approaches in the development of clinical, public health, or health systems guidelines’ (p. 1)	Global	Researchers
El-Harakeh et al. (2020)	‘To systematically identify and describe prioritization exercises that have been conducted for the purpose of the de novo development, update or adaptation of health practice guidelines’ (p. 1)	Global	Research institutions
Fadallah et al. (2020)	‘To systematically review the literature for proposed approaches and exercises conducted to prioritize topics or questions for systematic reviews and other types of evidence syntheses in any health-related area’ (p. 67)	Global	HCPs, researchers, patients, caregivers, general public
Garcia et al. (2015)	‘To systematically review literature on priorities in nursing research on health systems and services’ (p. 162)	Region of the Americas	Health institutions, universities, research centres, and practitioners
Garcia et al. (2017)	‘To identify and describe strategies to prioritize the updating of systematic reviews, health technology assessments or clinical guidelines’ (p. 11)	Global	Institutions
Graham et al. (2020)	‘To characterize research priority setting partnerships relevant to women’s health’ (p. 194)	Global	Women, HCPs
Hasson et al. (2020)	‘To identify and synthesize literature on international palliative care research priorities’ (p. 1)	HICs	Palliative care staff, healthcare professionals, patients, families, researchers, social care practitioners, service commissioners, policymakers, academics
Hawwash et al. (2018)	‘To review existing nutrition research priority-setting exercises, analyze how values are reported, and provide guidance for transparent consideration of values while setting priorities in nutrition research’ (p. 671)	HICs	HCPs, researchers, research institutes, experts in the field, dieticians, policymakers, family members, self-advocates, patients, Canadian Mental Health Association
Manafo et al. (2018)	‘To describe the evidence that exists in relation to patient and public engagement priority setting in both health ecosystem and health research’ (p. 1)	HICs	Health researchers and practitioners, patients, government agencies
McGregor et al. (2014)	‘To analyze all reported health research priority setting initiatives involving LMICs with a particular focus on methodologies’ (p. 2)	LMICs	Global or national or regional level populations
Mörelius et al. (2020)	‘To systematically identify the nature, range and extent of published pediatric nursing research priorities and synthesize them into themes’ (p.e57)	HICs	Nurses
Odgers et al. (2018)	‘To evaluate research priority setting approaches in childhood chronic diseases and to describe the priorities of stakeholders including patients, caregivers/families and health professionals’ (p. 943)	No setting specifications	Patients, family and caregivers, HCPs, policymakers
Roche et al. (2021)	‘To explore methodologies for identifying research priorities of the autism communities and whether research priorities identified by studies align across stakeholder groups’ (p. 337)	Global	Adults on autism spectrum, family members, professionals/practitioners, researchers, autism researchers
Rylance et al. (2010)	‘To systematically summarize priority topics for tuberculosis research from available publications and to describe how priorities were identified’ (p. 886)	Global	Experts, representative for patients, multidisciplinary international working groups
Reveiz et al. (2013)	‘To compare health research priority setting methods and characteristics among countries in Latin America and the Caribbean during 2002–2012’ (p. 1)	Latin America and the Caribbean	Government departments, researchers, policymakers, funders, NGOs
Rudan et al. (2017)	‘To review the first 50 examples of application of the CHNRI method, published between 2007 and 2016, and summarize the most important messages that emerged from those experiences’ (p. 1)	Global	Organizations/national bodies
Stewart et al. (2011)	‘To ascertain whether there is research literature to inform how patients and clinicians can work in partnership to identify and prioritize suggestions for research’ (p. 440)	Global	Patients and clinicians
Terry et al. (2018)	‘To see if the variation between reported research priorities can be overcome by a standardized mapping of the priorities against a common framework’ (p. 2)	No setting specifications	NR
Tomlinson et al. (2011)	‘To evaluate priority setting exercises that have taken place at national level in LMICs and recommend the constituents of a good priority setting process’ (p.2)	LMICs	National level priority setting with or without stakeholders
Tong et al. (2015)	‘To evaluate approaches to research prioritization in kidney disease and describe research priorities’ (p. 674)	Global	Patients, caregivers, HCPs or policymakers
Tong et al. (2017)	‘To evaluate research priority setting in solid organ transplantation and describe stakeholder priorities’ (p. 328)	Global	Transplant patients, caregivers, their HCPs, policymakers and researchers
Viergever et al. (2010)	‘To propose a checklist that outlines options for different approaches and defines nine common themes of good practice for health research prioritization processes’ (p.1)	Global	WHO and international research organizations experienced in health research priority setting
Wade et al. (2021)	‘To examine occasions of research priority setting in eating disorders’ (p. 346)	Global	Consumers who have lived experience of an eating disorder and their carers or support network
Yoshida (2016)	‘To understand the landscape of approaches, tools and methods used to prioritize health research and to assess their relative importance and applicability’ (p. 2)	Global	National and international bodies

CG: Clinical guideline; CHNRI: Child Health Nutrition Research Initiative; HCP: health care professional; HIC: high income country; HTA: health technology appraisal; JLA: James Lind Alliance; LMIC: low and middle-income country; MSK: musculoskeletal; OECD: Organisation for Economic Development and Co-Operation; NR: not reported; SCI: spinal cord injury; SR: systematic review

Populations included in the reviews varied, but typically would involve patients and their representatives, families and carers, researchers, clinicians, policymakers and research funders. The topics covered in the reviews varied from specific diseases or conditions, approaches for healthcare practice or research priority setting methods itself. Most studies reported process-related outcomes. Only one review (Odgers et al. 2018) included studies that provided sufficient details on implementation and evaluation to enable the reporting of outcome evaluations. None of the studies were able to provide evidence regarding changes in healthcare practice.

### Results of quality assessment

We used an adapted version of the AMSTAR 2 checklist for quality assessment. A full description of the results of the quality assessment are provided in “Appendix D”, and are also summarized here.

None of the included reviews were assessed to have high or moderate overall quality. Only two reviews (Tong et al. 2015; Cadorin et al. 2020) were assessed as low quality and the remaining 29 reviews were assessed to be of critically low quality overall. All the included reviews clearly reported the research questions and inclusion criteria for the review. However, more than 50% of the included reviews had the following issues that led to the low or critically low quality assessment:

- Did not report if the review protocol was available or report any deviations from the protocol;
- Did not extract data in duplicate, missing list of excluded studies with justifications for exclusions;
- Lack of using satisfactory technique for assessing the quality of individual studies and the research priority setting process;
- Did not report how sources of conflict of interest were managed in the included studies;
- Did not consider quality of the studies when performing the synthesis, or in the interpretation or the discussion sections; and
- Did not consider the impact of unpublished literature on the results of the priority setting process.

### Results of data synthesis

The results of the thematic analysis of the data synthesis are summarized below. Themes are organized by the main focus of the studies (i.e. engaging with stakeholders; methods; context; or health area) and further organized by geographical area and context (i.e. global, HICs, LMICs, specific countries/regions or no geographical specification) in recognition that the complexity of a priority setting process is dependent on the resources available.

### Identifying and engaging with stakeholders

#### Global

Two reviews (Stewart et al. 2011; Tong et al. 2017) showed that most studies reported collaborating with stakeholders, including patients, caregivers, physicians, allied health workers, researchers and policymakers to identify research questions. Stakeholders were engaged via surveys, telephone and face-to-face interviews, Delphi surveys and online and in-person forums. Tong et al. (2017) found that 32% (9/28) of studies identified differences between the priorities of patients/caregivers and health professionals and that only 4% (1/28) of studies indicated that stakeholder feedback was obtained and integrated into the proposed research priorities.

#### High-income countries

One review (Manafo et al. 2018) found that patient and public health involvement in setting research priorities resulted in subsequent studies focusing on these areas. However, operational details of the public involvement, cost, infrastructure and timelines are missing and make it difficult to replicate the process. The review found that local-level initiatives (versus regional or national) were most likely to ultimately impact patient-centredness and quality of care.

### Assessed all stages: focus on methods

#### Global

One review (Yoshida 2016) identified that the two most used approaches were the Child Health Nutrition Research Initiative (CHNRI) (26%, 43 of 165 studies) and Delphi methods (24%, 40 of 165). The other methods (in order of usage) were—expert consultation, literature reviews, James Lind Alliance (JLA), and online surveys. Health care providers and researchers were well represented in most initiatives reviewed; however, policymakers, funders, and affected populations were far less involved (Fadlallah et al. 2020). Few studies addressed translation of the priority topics into research questions or implementation and evaluation plans.

#### High-income countries

One review (Hawwash et al. 2018) assessed research priority setting methods in high-income countries and found that a diverse range of methods were used, including the Delphi method and CHNRI method. Many of the studies did not describe the stakeholders involved or the follow-up activities of the proposed activities.

#### Guidelines (clinical, public health, health systems)

Three reviews (El-Harakeh et al. 2019, 2020; Garcia et al. 2017) found that the most frequently reported criteria for prioritizing approaches for guideline development were the health burden of the disease; available evidence; potential impact of the intervention on health outcomes; and users’ interest. While health care providers were often involved in the prioritization exercises, very few involved patients. Generally, the methods for institutionalizing and implementing the prioritization processes were varied and poorly reported.

#### World Health Organization (WHO)

Two studies (Viergever et al. 2010; Terry et al. 2018) examined priority setting methods within the WHO. Terry et al. (2018) found that the most common research priority setting method was expert consultation, sometimes in conjunction with a literature review, but almost 70% of the identified research priorities were developed without using any additional criteria to rank the priorities with respect to potential health impact, feasibility or cost. Viergever et al. (2010) developed nine common themes of good practice in health research priority setting, which were categorized into three domains—preparation phase; methods for deciding upon priorities; and work performed after priorities have been set, which highlighted the importance of transparently reporting and disseminating the results of such work.

### Assessed all stages: focused on context

#### Region of the Americas

One review (Garcia et al. 2015) found that more than half of the priority setting documents did not have clear selection criteria and most did not have an implementation or evaluation plan.

#### High-income countries

Two reviews (Bryant et al. 2014; Hasson et al. 2020) highlighted the lack of consistent reporting and evaluation of priority setting processes and recommended that a multi-disciplinary advisory group should oversee the priority setting process; broad representation of stakeholders is critical; objective, clearly defined criteria should guide the generation of priorities; and the impact of the priority setting processes should be evaluated. Specific focus needs to be placed on elevating the voice of patients to enhance the validity of identified priorities (Hasson et al. 2020).

#### Low and middle-income countries

Four reviews (Badakhshan et al. 2018; McGregor et al. 2014; Tomlinson et al. 2011; Reveiz et al. 2013) recommended incorporating mechanisms for disseminating priority setting results, creating implementation plans and processes for revising priorities, and engaging stakeholders. Importantly, these reviews suggested establishing a communication channel with neighbouring countries about the priority setting process. The reviews recommended establishing regional health research agendas, harmonizing research setting approaches to enable greater comparability and strengthening collaboration between groups of researchers sharing the same interests.

#### No geographic specification

Rudan et al. (2017) did not have a specific geographic focus but examined studies that specifically used the CHNRI method. It found that from 2016 onwards it was often adapted to suit respective contexts. It was used in a range of different health fields due to its: systematic and democratic nature, acceptable framework to handle many research questions, transparency and replicability, clear definition of the context and priority setting criteria, adaptability, and ease of conduct.

### Assessed all stages: focus on health area

#### High-income countries

Three reviews (Booth et al. 2018; Cadorin et al. 2020; Mörelius et al., 2020) found that consensus building methods, such as Delphi processes, multi-staged surveys and focus groups, were commonly used to develop priorities. However, a key limitation of many studies was the lack of engagement with patients and carers or professional groups outside of healthcare sectors, such as social workers and teachers, in the research prioritization exercise. Where sought, only minimal involvement was secured. This was concerning given that priorities of patients often differ from those of clinicians and the priority setting processes did not reflect on whether the priorities identified reflected the priorities of the key consumers of the healthcare services (Booth et al. 2018; Cadorin et al. 2020; Mörelius et al., 2020). Integrating qualitative studies involving patients in focus or discussion groups to share the results of the literature and establish with them the main priorities would be a starting point to develop a new research priority agenda that can be adapted locally according to each context (Cadorin et al. 2020). This would involve going beyond lists of priorities to explore what these priorities mean for patients and consumers. In addition, greater collaboration with other professionals is desirable (Cadorin et al. 2020).

Two studies (Cadorin et al. 2020; Mörelius et al., 2020) discussed practical aspects of priority setting processes. Considering the economic and financial context in which the priority setting processes are situated is important for increasing the likelihood that research priorities will be taken forward. Additionally, Mörelius et al. (2020) was the only study that highlighted the need to improve the actual implementation of evidence into practice.

#### Global

Commonly used methods for developing priorities included surveys, interviews or focus groups with consumers and stakeholders (Alqahtani et al. 2021; Roche et al. 2021). Methods for recruiting participants included through support groups, research networks, websites, social media, special interest groups, conferences and contacts of the research team (Roche et al. 2021).

Seven reviews (Bourne et al. 2018; Bragge et al. 2015; Graham et al. 2020; Odgers et al. 2018; Roche et al. 2021; Rylance et al. 2010; Tong et al. 2015) recommended that future research priority setting initiatives have a clear aim, use robust methods and include all relevant stakeholders. Future studies could improve the robustness of methods by utilizing good practice guidelines in research and reporting of priority setting, such as those proposed by the JLA, WHO (2020a) or Viergever et al. (2010). Integrated research strategies that incorporate a co-design or participatory research perspective have the potential to drive better outcomes (Roche et al. 2021; Wade et al. 2021). Consumers should be involved at every stage of the priority setting process, especially when determining how to implement the priorities to improve future practice (Wade et al. 2021). Roche et al. (2021) underscored this need by questioning whether priorities identified by healthcare professionals, researchers etc. are truly reflective of the target communities and people with lived experience. Using a ‘bottom up’ approach where consumers can express their priorities in their own words would increase the relevance of priorities (Roche et al. 2021).

Strategies for implementing, institutionalizing and measuring the impact of the research priorities included liaising with key stakeholders, disseminating priorities through key organizations and monitoring the impact of priorities on grant applications and grants awarded (Odgers et al. 2018). Bragge et al. (2015) reported that the findings from their review subsequently informed the development of a regional research strategy.

Notably, two of the most recently published reviews (Roche et al. 2021; Wade et al. 2021) highlighted that translation of research priorities across community or international settings will also need to account for societal and cultural differences.

## Discussion

### Summary of main findings

This study identified, appraised and synthesized the systematic reviews in the recent literature on research priority setting to propose a draft evidence-informed framework for countries to consider while developing their national research agendas. The findings of our review reflected those in other systematic reviews in the field. Like other evidence syntheses (Hanney et al. 2020), this overview highlighted the value and importance of establishing evidence-informed priority setting structures for the development of research agendas. Involving a diverse range of stakeholders in the priority setting process can ‘increase the legitimacy, credibility, transparency, and acceptability of the identified priorities’ (Fadlallah et al. 2020). However, engaging with stakeholders is challenging in terms of resources, capacity and feasibility (Fadlallah et al. 2020).

As was found by Sharma et al. (2018) and others, many priority setting documents do not clearly or consistently report methods (Garcia et al. 2015; Bryant et al. 2014; Hasson et al. 2020). Commonly used priority setting approaches included formalized methods such as the CHNRI and Delphi approaches, and informal approaches, such as expert consultation. Having an implementation and evaluation strategy could assist in translating the results of the priority setting exercises into research (Manafo et al. 2018; Tong et al. 2017).

The WHO has recently reviewed and updated methodologies related to health research priority setting and developed guidance for its staff and the research priority setting exercises for which the WHO has responsibility (World Health Organization 2020a). The Guide, *A Systematic Approach for Undertaking a Research Priority-Setting Exercise*, contains a framework for developing research priority setting exercises. This Guide, which was initially developed for use amongst WHO Headquarter staff, appears to be mainly based on good practice examples and methodologies already in use across the WHO. In comparison, our overview used a systematic process to identify literature published within the last decade on health research priority setting methods and used a transparent and reproducible approach to create a framework.

**Fig. 2:**
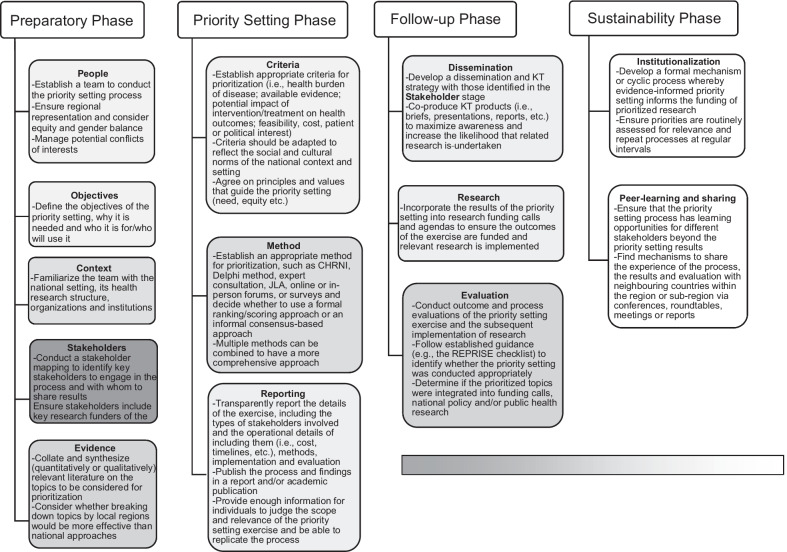
Priority setting framework. The gradient of grey reflects the frequency with which domains have been considered in existing frameworks, with dark grey representing frequent consideration to white highlighting novel elements identified in the current overview. CHNRI: Child Health Nutrition Research Initiative; JLA: James Lind Alliance; KT: knowledge translation

Our framework draws from four existing frameworks (Fadlallah et al. 2020; Nasser et al. 2020; Viergever et al. 2010; World Health Organization 2020a) and incorporates evidence from the thematic analysis of the included reviews to create a comprehensive approach for countries to develop future research agendas. The priority setting framework (Fig. 2) demonstrates the preliminary framework with four key phases: (1) preparatory, (2) priority setting, (3) follow-up phase and (4) sustainability phase.

### Preparatory phase

Four existing frameworks (Fadlallah et al. 2020; Nasser et al. 2020; Viergever et al. 2010; World Health Organization 2020a) note the importance of including relevant stakeholders in the process and having a participatory approach, which is also valued as a key component in most of the included reviews in our study. Additionally, our overview highlighted the importance of having a bottom-up approach where researchers work with consumers in a co-design and co-production process so that resulting priorities are reflective of patient needs (Cadorin et al. 2020; Roche et al. 2021; Wade et al. 2021). Only one of the previous frameworks (Nasser et al. 2020) highlights the importance of having a dedicated team to lead the priority setting process.

While only one of the previous frameworks (World Health Organization 2020a) highlighted the need to outline the objective of the priority setting process, all our included reviews indicated the objective of their review, which was then used to guide their inclusion of their primary studies and synthesis. Many of the included reviews also discussed the need for a formal search of the background literature to situate the priority setting within the current literature base and this contextualization was also noted by all four of the existing frameworks (Nasser et al. 2020; Viergever et al. 2010; Fadlallah et al. 2020; World Health Organization 2020a),

### Priority setting phase

Our overview found that included studies usually used a combination of mechanisms, such as surveys, workshops and expert consultation, often as part of formal processes for determining priorities, such as CHNRI, Delphi and JLA methods (Cowan and Oliver 2013; Fadlallah et al. 2020; Yoshida 2016). The existing frameworks do not clearly outline what methods should be followed when setting priorities, nor do they discuss the importance of integrating qualitative with quantitative data to understand the reasoning behind stakeholders’ rankings (Roche et al. 2021; Wade et al. 2021). The qualitative evidence gained through focus groups and interviews can provide valuable insights into the lived experiences of patients and consumers to contextualize quantitative rankings of priorities and should be included into processes (Roche et al. 2021; Wade et al. 2021).

The importance of reporting the findings is seen in two existing frameworks (Nasser et al. 2020; World Health Organization 2020a). From our overview we know that there is a lack of consistent reporting and the authors of the reviews recommend having multi-disciplinary advisory groups to oversee the whole process (Bryant et al. 2014; Hasson et al. 2020). It was also noted that often the costs, mechanisms and details of patient and public participation are not reported, making it difficult for replication (Manafo et al. 2018), though some advice around recruitment is available from one review (Roche et al. 2021). We also suspect the presence of non-reporting, in addition to poor reporting, which could be improved by following guidance for appropriate, systematic and transparent reporting of priority setting practices, such as the REPRISE checklist (Tong et al. 2019).

### Follow-up phase

Two of the existing frameworks (Fadlallah et al. 2020; World Health Organization 2020a) and our overview discuss the importance of having a structured dissemination strategy that shares the priorities with key organizations and stakeholders to facilitate their subsequent implementation (Badakhshan et al. 2018; McGregor et al. 2014; Tomlinson et al. 2011; Reveiz et al. 2013; Odgers et al. 2018). Our overview also highlighted that the translation of these research priorities should account for societal and cultural differences as they are translated across different communities and settings (Roche et al. 2021; Wade et al. 2021). The evaluation of the priority setting process was considered in all four of the existing frameworks (Nasser et al. 2020; Viergever et al. 2010; Fadlallah et al. 2020; World Health Organization 2020a) as it was in our included studies (Bryant et al. 2014; Hasson et al. 2020). Two of the frameworks (Nasser et al. 2020; World Health Organization 2020a) consider some elements of research funding cycles within their feedback and evaluation domains. Our overview noted that the impact of priorities should be monitored by assessing whether priorities are reflected in grants being awarded (Bragge et al. 2015; Odgers et al. 2018).

### Sustainability phase

The most notable difference between our framework and that of WHO (2020a) is our sustainability phase, which includes considerations for developing mechanisms for institutionalizing the priority setting process into the research cycle and creating peer-learning amongst colleagues and regions. This element of sustainability is crucial for ensuring that policymaking is underpinned by prioritized research and that the priority setting process can build a wider community of practice. One included review (Odgers et al. 2018) highlights the value of institutionalization (i.e. ensuring that priority setting processes are cyclical, as opposed to singular, events within an organization). Two reviews (Cadorin et al. 2020; Mörelius et al., 2020) also noted the importance of economic aspects of priority setting processes to ensure that an environment or context is made whereby research priorities are taken forward. The four LMIC reviews (Badakhshan et al. 2018; McGregor et al. 2014; Tomlinson et al. 2011; Reveiz et al. 2013) recommended establishing communication channels with neighbouring countries to share best practices, establish regional health research agendas where possible, harmonize priority setting processes to enable greater comparability and strengthen collaboration.

### Methodological strengths and limitations

A rapid review approach requires that components of the systematic review process are simplified or omitted to produce information in a timely manner and are increasingly used when facing resource and time constraints (Tricco et al. 2015). One of the limitations of this overview is that we only searched in two databases. However, the searches and the experts we contacted in our network identified all the relevant studies included in this overview. Due to the limited use of indexing for studies related to priority setting, a broader search strategy would have increased the number of irrelevant results to a degree that the review would have been too resource intensive to complete. Moreover, there is no indication that our approach to searching introduced any meta-biases. While it is theoretically possible that additional studies may have been conducted but not yet published, or that additional studies may not have been identified, this is unlikely.

While we did not conduct an extensive search of the grey literature as this was outside of the scope of our rapid review, our search in the WHO IRIS database captured relevant WHO reports, which is the most relevant grey literature for the context of our review. We also searched the reference lists of included studies to ensure we had captured all relevant reviews. Additionally, several of the included studies in our review, such as Viergever et al. (2010) included a search of the grey literature. Though study selection was not completed fully in a double-blind manner, the authors performed a blinded-dual screen of a portion of the studies to ensure a high degree of agreement (> 80%) before completing the unblended screening on the rest of the records.

A key strength of this study is that this is the first review of its kind that the authors are aware of that uses systematic review evidence to underpin and develop a framework for national research priority setting in a systematic and transparent manner. Notably, this review also created an adapted AMSTAR tool, which enabled us to perform a quality assessment. There are currently no standard methods for conducting priority setting methods, so our overview provides an assessment of the most recently used methods and offers guidance on the conduct of a systematic review of priority setting methods.

### Future research

Future studies should confirm and consolidate the framework developed in this review with relevant stakeholders. Further work and validation would be needed to ensure its applicability and appropriateness at a national level. A process evaluation could help assess the extent to which the priorities generated have informed research or research funding, or the extent to which they have been adopted into the field. More research is needed into the practicalities of engaging stakeholders in priority setting exercises.

### Differences with protocol

The authors intended to base the data extraction form on consolidated reporting items identified in the REporting guideline for PRIority SEtting of health research (REPRISE) checklist (Tong et al. 2019), but instead used the framework developed by Nasser et al. (2020), which the data fit better. After our overview was completed, the WHO headquarters published their priority setting guidance, *A Systematic Approach for Undertaking a Research Priority-Setting Exercise* (World Health Organization 2020a). To acknowledge this report and remain transparent, we compared our findings and recommendations with those they recommended in the Discussion section.

## Conclusions

Research priority setting is important for strengthening health systems and evidence-informed policies; countries and regions need to invest further within this field. As health systems experience continued pressures and constraints, it is essential that health research funds are allocated in an evidence-informed, transparent and structured manner. Our overview of reviews has reconfirmed aspects of existing frameworks, but has also identified new concepts that we have integrated either as new elements or revisions to existing ones. Notably, our overview has identified that the integration of priority setting exercises into local, national or international level decision making can support sustainability as, currently, some key lessons on how to set priorities for research get lost in administrational, organizational and staff changes. Our overview presents a draft framework and methodological tools to support countries in answering questions that are of value to society and whose outcomes inform policies that improve the health and well-being of populations.

## Data Availability

The datasets supporting the conclusions of this article are included within the article and its additional files.

## Appendix A: Search strategy and summary of search results

Search Strategy OVID (MEDLINE) 1 April 2020.

**Table:**

	Searches	Results	Type
1	"Review"/	2589030	Advanced
2	(research adj3 (priorit$ or agenda or strateg$)).kw,ti	5213	Advanced
3	(review or analysis or map* or database).ab,kw,ti	5557460	Advanced
4	3 or 1	7064197	Advanced
5	4 and 2	1911	Advanced
6	from 5 keep 1–1911	1911	Advanced

Search Strategy OVID (MEDLINE) 29 May 2021

**Table:**

	Searches	Results	Type
1	"Review"/	2756172	Advanced
2	(research adj3 (priorit$ or agenda or strateg$)).kw,ti	5676	Advanced
3	(review or analysis or map* or database).ab,kw,ti	6124712	Advanced
4	1 or 3	7683993	Advanced
5	2 and 4	2102	Advanced
6	limit 5 to (english language and yr = "2020–2021")	211	Advanced

WHO IRIS search strategy (14/04/2020)

**Table:**

	Searches	Results of search on 14/04/2020	Results of search on 1 June 2021
1	“research priority setting”	218	15
2	“research agenda setting”	27	6

Summary of searches:

**Table:**

	Database	Results of first search	Results of updated search	Total	Total after removal of duplicates
1	MEDLINE via OVID	1911	211	2122	1734
2	WHO IRIS	245	21	266	
3	Other sources	7	-	7	

## Appendix B: Adapted AMSTAR-2 for use with reviews of priority setting

### Adaptation developed by Dr Mona Nasser

- **Item 1: Did the research questions and inclusion criteria for the review make it clear what type of research priority setting exercises they are focusing on?**
  - For yes—If the description if clear.
  - For no—If the description is not clear.
- **Item 2: Did the report of the review contain an explicit statement that the review methods were established prior to conduct of the review and did the report justify any significant deviations from the protocol?**
  - For yes—If there is a protocol available and reported.
  - For no—If there is not available or not reported.
- **Item 3: Did the review authors explain their selection of the study designs for inclusion in the review?**
  - For yes—If the authors describe and differentiate different type of studies and their implications in the review. Most these types of reviews just include any reporting of research priority setting without considering whether they have done evaluation or not.
  - For no—If the choice of the study design for evaluation is no appropriate for the objective.
- **Item 4: Did the review authors use a comprehensive literature search strategy appropriate for the research question and context of the study?**
  - For yes—If they are looking at all priority setting in an organisation (for example, WHO or Cochrane) and have demonstrated they have taken an appropriate strategy to find all studies. If it is a review of published peer-reviewed literature, it will follow the standard AMSTAR rules, that is, if at least two bibliographic databases were searched and provided key words and publication restrictions.
  - For partial yes—If they are only look at priority setting in an organisation and they have used a selection of the reports of that organisation.
  - For no—If no systematic approach to searching the literature has been outlined.
- **Item 5: Did the review authors perform study selection in duplicate?**
  - For yes—At least two reviewers independently agreed on selection of eligible studies and achieved consensus on which studies to include OR two reviewers selected a sample of eligible studies and achieved good agreement (at least 80 percent), with the remainder selected by one reviewer.
  - For no—Study selection not completed in duplicate.
- **Item 6: Did the review authors perform data extraction in duplicate?**
  - For yes—At least two reviewers achieved consensus on which data to extract from included studies or two reviewers extracted data from a sample of eligible studies and achieved good agreement (at least 80 percent), with the remainder extracted by one reviewer.
  - For no—Data extraction not performed in duplicate.
- **Item 7: Did the review authors provide a list of excluded studies and justify the exclusions?**
  - For yes—Authors provided a list of excluded studies and justified the exclusions.
  - For partial yes—Authors provided a list of all potentially relevant studies that were read in full-text form but excluded from the review.
  - For no—Authors did not provide a list of excluded studies or justifications for the exclusion.
- **Item 8: Did the review authors describe the included studies in adequate detail?**
  - For yes—If they provide details of the research priority setting exercises.
  - For partial yes—If they mentioned what type of research priority setting was included.
  - For no—If they did not provide adequate details or descriptions of the included studies.
- **Item 9: Did the review authors use a satisfactory technique for assessing the quality of individual studies and the research priority setting processes in the review.**
  - For yes—If they have a process in place and described and used it.
  - For no—If no technique for assessing the quality of individual studies is described or implemented.
- **Item 10: Did the review authors report on the sources of funding for the studies included in the review?**
  - For yes—Must have reported on the sources of funding for individual studies included in the review. Reporting that the reviewers looked for this information but it was not reported by study authors also qualifies.
  - For no—Did not report on the sources of funding for individual studies.
- **Item 11: Did the authors use an appropriate approach to synthesize the information?**
  - For yes—If they used a systematic and critical approach to evaluate the studies—this might be statistical or narrative or a combination of both.
  - For no—If no approach was outlined for synthesizing the information.
- **Item 12: Did the synthesis process consider the quality of the studies when combining?**
  - For yes—If they have considered the quality of the studies when performing the synthesis.
  - For no—If they have not considered the quality of the studies when performing the synthesis.
- **Item 13: Did the interpretation and discussion of the results of the review considered the quality of the individual priority setting process or the evaluation study?**
  - For yes—If the interpretation and discussion considered the quality of the individual studies.
  - For no—If the interpretation and discussion did not consider the quality of the individual studies.
- **Item 14: Did the review authors provide a satisfactory explanation for, and discussion of, any major discordance or differences in the results of the priority setting exercise or the evaluation of them?**
  - If you see some major differences either in the final priorities or how the process is implemented or evaluation results, the authors need to explain and rationalize them in the synthesis process.
  - For yes—There was no significant heterogeneity in the results OR if heterogeneity was present, the authors discussed the impacts on the results.
  - For no—If heterogeneity is present and they do not explain or report it.
- **Item 15: Did they consider the impact of unpublished literature on the results?**
  - For yes—If they discuss and consider the impact of unpublished literature on the results and interpretation.
  - For no—If they do not discuss and consider the impact of unpublished literature on the results and interpretation.
- **Item 16: Did the review authors report any potential sources of conflict of interest, including any funding they received for conducting the review?**
  - For yes—The authors reported no competing interests OR The authors described their funding sources and how they managed potential conflicts of interest.
  - For no—The authors did not report whether there were competing interests, conflicts of interest or funding sources.

## Appendix C: Excluded studies

**Table:**

References	Reasons for exclusion
Abramowitz et al. (2018)	Core concept does not evaluate research priority setting exercises
Anstee et al. (2011)	Core concept does not evaluate research priority setting exercises
Barnes et al. (2015)	Core concept does not evaluate research priority setting exercises
Bassetti et al. (2015)	Core concept does not evaluate research priority setting exercises
Bragge et al. (2015)	Core concept does not evaluate research priority setting exercises
Buchholz et al. (2018)	Ineligible study design, i.e. not a critical or systematic review
Consortium from Altarum (2012)	Ineligible study design, i.e. not a critical or systematic review
Crewdson et al. (2018)	Ineligible study design, i.e. not a critical or systematic review; Core concept does not evaluate research priority setting exercises
Cumpston et al. (2012)	Core concept does not evaluate research priority setting exercises
Davis et al. (2015)	Core concept does not evaluate research priority setting exercises
Delost and Nadder (2014)	Ineligible study design, i.e. not a critical or systematic review; Core concept does not evaluate research priority setting exercises
Doosti-Irani and Holakouie-Naieni (2016)	Core concept does not evaluate research priority setting exercises
Fabbri et al. (2018)	Ineligible study design, i.e. not a critical or systematic review; Core concept does not evaluate research priority setting exercises
Foster et al. (2018)	Core concept does not evaluate research priority setting exercises
Hasson et al. (2020)	Duplicate
Hill et al. (2019)	Ineligible study design, i.e. not a critical or systematic review; Core concept does not evaluate research priority setting exercises
Knight et al. (2014)	Core concept does not evaluate research priority setting exercises
Johnston et al. (2016)	Ineligible study design, i.e. not a critical or systematic review; Core concept does not evaluate research priority setting exercises
Jones and Geneau (2012)	Core concept does not evaluate research priority setting exercises
Karimkhani et al. (2016)	Core concept does not evaluate research priority setting exercises
Kong et al. (2019)	Core concept does not evaluate research priority setting exercises
Kühne et al. (2021)	Core concept does not evaluate research priority setting exercises
Morton et al. (2012)	Core concept does not evaluate research priority setting exercises
Nicolau et al. (2012)	Core concept does not evaluate research priority setting exercises
Okland et al. (2017)	Core concept does not evaluate research priority setting exercises
Oncology Nursing Forum (2019)	Ineligible study design, i.e. not a critical or systematic review
Pozzar and Berry (2017)	Core concept does not evaluate research priority setting exercises
Pratt (2020)	Ineligible study design, i.e. not a critical or systematic review
Sebastianski et al. (2019)	Core concept does not evaluate research priority setting exercises
Sigfrid et al. (2019)	Core concept does not evaluate research priority setting exercises
Van Royen et al. (2010)	Core concept does not evaluate research priority setting exercises
Von Ah et al. (2019)	Ineligible study design, i.e. not a critical or systematic review
Wald et al. (2014)	Ineligible study design, i.e. not a critical or systematic review; Core concept does not evaluate research priority setting exercises
World Health Organization (2011)	Ineligible study design, i.e. not a critical or systematic review
World Health Organization (2020a, b)	Ineligible study design, i.e. not a critical or systematic review
World Health Organization (2021)	Ineligible study design, i.e. not a critical or systematic review
Woud et al. (2017)	Core concept does not evaluate research priority setting exercises
Wykes et al. (2015)	Ineligible study design, i.e. not a critical or systematic review

## Appendix D: Quality assessment

We used adapted version of AMSTAR criteria for quality assessment with 16 items. None of the included reviews were assessed to have high or moderate overall confidence in the results. Only two reviews (Tong et al. 2015; Cadorin et al. 2020) were assessed as low quality and the remaining 29 reviews were assessed to have critically low-quality overall confidence.

All the 29 included reviews clearly reported the research questions and inclusion criteria for the review. Eighteen included reviews did not report if the review protocol was available or report any deviations from the protocol (Alqahtani et al. 2021; Badakhshan et al. 2018; Bourne et al. 2018; Bragge et al. 2015; Bryant et al. 2014; Garcia et al. 2015; Hasson et al. 2020; McGregor et al. 2014; Odgers et al. 2018; Reveiz et al. 2013; Rudan et al. 2017; Terry et al. 2018; Tomlinson et al. 2011; Tong et al. 2015; Tong et al. 2017; Yoshida 2016, Wade et al. 2021). Nine reviews did not explain their selection of the study designs for inclusion (Badakhshan et al. 2018; Booth et al. 2018; Garcia et al. 2015; Hasson et al. 2020; Odgers et al. 2018; Rudan et al. 2017; Rylance et al. 2010; Yoshida 2016).

Only four reviews did not use a comprehensive literature search strategy (Roche et al. 2021; Viergever et al. 2010; Wade et al. 2021; Yoshida 2016). In 13 out of 29 reviews, the review authors had selected the studies in duplicate (Booth et al. 2018; Bourne et al. 2018; Bragge et al. 2015; Cadorin et al. 2020; El-Harakeh et al. 2019; El-Harakeh et al. 2020; Fadlallah et al. 2020; Garcia et al. 2017; Graham et al. 2020; Mörelius et al. 2020; Stewart et al. 2011; Rylance et al. 2010; Tong et al. 2017). Fifteen reviews did not extract data in duplicate (Alqahtani et al. 2021; Bragge et al. 2015; Garcia et al. 2015; Manafo et al. 2018; McGregor et al. 2014; Reveiz et al. 2013; Roche et al. 2021; Rudan et al. 2017; Stewart et al. 2011; Terry et al. 2018; Tomlinson et al. 2011; Viergever et al. 2010; Wade et al. 2021; Yoshida 2016).

Only four reviews provided a list of excluded studies with justifications for exclusions (Bourne et al. 2018; Fadlallah et al. 2020; Garcia et al. 2017; Rylance et al. 2010) and one review mentioned that the list of excluded studies can be requested to the review authors (Booth et al. 2018). Only one review did not describe the included studies in adequate detail (Wade et al. 2021) and three studies just mentioned what type of research priority setting was included (Graham et al. 2020; Manafo et al. 2018; Terry et al. 2018). Fourteen studies used satisfactory technique for assessing the quality of individual studies and the research priority setting process in the review. Only eight studies reported how sources of conflict of interest were managed in the included studies (Booth et al. 2018; Bourne et al. 2018; El-Harakeh et al. 2020; Fadlallah et al. 2020; Odgers et al. 2018; Reveiz et al. 2013; Terry et al. 2018; Tong et al. 2015). Majority of the included studies used an appropriate approach to synthesis the information except for three (Graham et al. 2020; Manafo et al. 2018; Yoshida 2016) which did not outline the information on data synthesis. Only one study considered the quality of the studies when performing the synthesis (Cadorin et al. 2020).

Six studies did the interpretation and discussion of the results considering the quality of the priority setting process or the evaluation of the study (Cadorin et al. 2020; Graham et al. 2020; Manafo et al. 2018; Mörelius et al. 2020; Odgers et al. 2018; Tong et al. 2015). Six studies did not provide any satisfactory explanation or discussion of major discordance in the results of the priority setting exercise (Badakhshan et al. 2018; Rylance et al. 2010; Viergever et al. 2010; McGregor et al. 2014; Bragge et al. 2015; Garcia et al. 2015; Graham et al. 2020). The impact of unpublished literature on the results of the review was considered only in six of the included studies (Alqahtani et al. 2021; Bourne et al. 2018; Cadorin et al. 2020; Garcia et al. 2017; Roche et al. 2021; Tong et al. 2015). Review authors of four studies did not report any potential sources of conflict of interest including any funding they received for conducting the review (Garcia et al. 2015; El-Harakeh et al. 2020; Garcia et al. 2017).

## Abbreviations

AMSTAR 2: A MeaSurement Tool to Assess systematic Reviews 2
CHNRI: Child health nutrition research initiative
HIC: High-income country
IRIS: Institutional repository for information sharing
JLA: James Lind alliance
LMIC: Low- and middle-income country
PRESS: Peer-Review of Electronic Search Strategies
PRISMA-P: Preferred reporting items for systematic reviews and meta-analysis protocols
REPRISE: Reporting guideline for PRIority Setting
WHO: World health organization
